# Supplementary material for: Effect of gas exchange data selection methods on resting metabolic rate estimation in young athletes
Source: PLoS One. 2023 Sep 20;18(9):e0291511. doi: 10.1371/journal.pone.0291511 (PMC10511082; doi:10.1371/journal.pone.0291511)
Supplement: Attachment — Submitted filename: Response to the Reviewers.docx [file pone.0291511.s003.docx]

Dear editors and reviewers.,

The authors would like to thank you for these much relevant comments and suggestions, which contribute to improving the quality of our paper. After meticulously analyzing the comments and questionings, the errors that were pointed out, and the suggestions that were sent to us, we modified it to improve the writing and the overall understanding of our paper. We hereby send the explanations and modifications related to the comments we received. All modifications in the manuscript were tagged in yellow in file with track changes.

**Abstract**

Q1. In the objectives, the authors mention "shortening protocol". I believe the authors meant protocol shortening. Please, check it.

A: Thank you. We modify it following your consideration.

**Introduction**

Q2. [Page 3 - Lines 50-52]: This sentence sounds redundant. "…have increased energy needs than sedentary individuals because they have greater energy demands." Please, rephrase or be more specific.

A: We modified it to be more specific to energy needs due to exercise training. The sentence, after modification, is “...have higher energy needs than sedentary individuals because training promotes higher energy demands”.

Q3. [Page 3 – Line 60]: A period (full stop) is missing after Weir, 1949. Please, check it.

A: We insert a period after Weir, 1949. Thank you.

Q4. [Introduction – 2nd paragraph]: The second paragraph is too long. I suggest breaking it down into 2 or 3 paragraphs. Maybe, lines 65 and 81 may be split into new paragraphs. Please, reconsider the length.

A: As suggested, we splitted the second paragraph in 3.

Q5. [Overall Introduction]: The Introduction is well written and gathers good evidence to lead authors to the aimed gap. However, it is still unclear why the sample effect would affect the signal nature and confirm the originality besides the previous studies that have already compared the different techniques. Please, consider improving the gap elucidation.

A: Thank you for your question. We insert the following sentence in the introduction. “...given that it is known that VO2 and VCO2 kinetics could be influenced by gender, age and health status (Alcantara et al., 2022)”

**Methods**

Q6. [Page 5 - Lines 96-97]: 12 to 18 years is a wide gap. Is any assessment of maturation included in sample profiling?

A: As the visual assessment using Tanner's Maturation Scale is considered the most reliable protocol for evaluating maturation, and due to ethical considerations in Brazil requiring assessment by a Pediatric Doctor, we did not include maturation evaluation in our samples. We considered this as a limitation in the last paragraph of the discussion.

Q7. [Page 6 – Line 132-133]: Was the equipment set to collect every minute, or was a fixed time averaging of 1 min was set as standard output? Please, specify it.

A: The equipment collected one sample each 1 minute. We describe it in Methods.

Q8. [Page 6 – Line 148-149]: "(…) values within the filtering intensities", you mean that it met the thresholds or ranges, right? Please, adjust it.

A: We adjusted the text to make it clearer.

Q9. [Page 7 – Line 150-151]: Instead of "it was computed the first…", please adjust the sentence like "The first and the best SSt were computed as previously proposed…".

A: It was adjusted as suggested.

Q10. [Page 7 – Line 150-156]: Please, gather all SSt detail in the same paragraph (e.g., between TI and Filtering or after Filtering).

A: It was adjusted as suggested.

Q11. [Page 7 – Line 155-156]: Please, cite the Weir equation reference in addition to Hansen et al. (1998).

A: We exchanged Hansen et al. by Weir et al..

Q12. [Page 8 – Line 162-165]: Could authors further explain this ANOVA choice and the invalid values issues? It was not clear, and readers may not follow these missing values.

A: Possibly there was a confounding use of statistical terms here. GraphPad Prism calls Mixed ANOVA when there are missing values in the matrix for ANOVA’s calculations. In this case, the software fitting the data to insert the “missing” values and run the repeated measures ANOVA. This strategy was used instead of excluded the valid data (calculated using other data selection methods) for the subject. Only TI6-10 and 10minSSt returned missing data and we reported it in the text to let it clear to the reader. We preferred to exclude the term “mixed” in the text to eliminate a possible confusion about the statistical terms. Finally, please see the following link link (<https://www.graphpad.com/guides/prism/latest/statistics/stat_missing-values-in-repeated-mea.htm>) with the explanation regarding the missing values in repeated measures ANOVA.

**Results**

Q13. [Table 1]: Please, replace all decimal commas with dots and standardize the decimal places for each variable.

A: The values were adjusted as suggested.

Q14. [Figures 1 and 2]: Please include the effect sizes for all comparisons.

A: The effect sizes were included as suggested.

Q15. [Figures 1 and 2 – and Methods]: It was unclear if the authors included the Best or First SST methods in this analysis. I needed to read several times the methods section, and it is still not totally clear after it was broken down for Figure 3. Were methods combined here? Please, improve the explanation or include another name for the combined approach.

A: We made a minor adjustment in the statistical section trying to let it clearer.

Q16. [Page 9 – Line 187]: I believe the authors meant to cite subfigure C instead of B. Please, adjust it.

A: Thank you. It was amended.

Q17. [Page 9 – Lines 193-194]: I believe the authors meant to cite subfigure B instead of C. Please, adjust it.

A: Thank you. It was amended.

Q18. [Page 9 – Lines 205-208]: Figure 2 only presents CVs' mean (sd). The occurrence of the achievement of CV limits was not plotted. Please, consider including or replacing it with a dispersion illustration highlighting this occurrence or reconsider the sentence.

A: We made a minor adjustment in the statistical section trying to let it clearer.

Q19. [Page 10 – Line 215]: There is an incomplete parathesis between "SEE" and "of approximately…". Please, adjust it.

A: Thank you. It was adjusted.

Q20. [Page 10 – Line 217]: You have cited Table 3 twice. Please, adjust it.

A: Thank you. It was adjusted.

Q21. [Page 10 – Line 217]: I think the authors missed the "Figure 3 Here" indication after the last results paragraph.

A: Thank you. It was adjusted.

**Discussion**

Q22. [Overall Discussion]: Please, review the citing style [e.g., In contrast to Alcantara et al. (Alcantara et al., 2020), (...)]. It seems redundant and repeats several times across the paragraphs. In Line 272, brackets are used "(…) Freire et al. [14]".

A: Thank you. It was adjusted in the whole discussion.

Q23. [Discussion - 2nd and 3rd Paragraphs]: Please, elucidate how populations may interfere with the signal nature. In addition, I am concerned if differences can be attributed to populations solely or the metabolic carts brand and, maybe more determinately, the signal sampling adopted.

A: Thank you for your question. We wrote a new paragraph before 8th paragraph elucidating how populations may interfere signal nature and, thus, RMR estimation.

“It is also important to highlight the real need to assess RMR in adolescent athletes. It is known that the kinetics of VO2 and VCO2 can be influenced by health status, gender, and age (Alcantara et al., 2022). Therefore, it is important to identify the optimal method for selecting gas exchange data to estimate RMR in this young population. A method that reduces intra-assessment variability, reflected by lower CVs for VO2, VCO2, RER, and RMR, would minimize the risk of methodological error and enhance the accuracy of RMR estimation. Considering that RMR is the main component of total energy expenditure, it is necessary to establish differentiated techniques for gas exchange analysis for this measurement.“

Q24. [Discussion – 6th paragraph]: Please, also mention the populations when comparing the studies to improve readers' understanding.

A: Thank you. It was adjusted.

Q25. [Discussion – 8th Paragraph]: The signal sampling of 1 min can also limit the assessment of the methods. Please, consider discussing it.

A: Thank you. The sentence below was added in 9th (old 8th) paragraph.

“Data acquisition method (one sample per minute) may increase methodological error and it is necessary to evaluate the methods of selecting gas exchange analysis further using breath-by-breath sample collection method.
